# Semantic Similarity for Automatic Classification of Chemical Compounds

**DOI:** 10.1371/journal.pcbi.1000937

**Published:** 2010-09-23

**Authors:** João D. Ferreira, Francisco M. Couto

**Affiliations:** LaSIGE, University of Lisbon, Lisbon, Portugal; University of St Andrews, United Kingdom

## Abstract

With the increasing amount of data made available in the chemical field, there is a strong need for systems capable of comparing and classifying chemical compounds in an efficient and effective way. The best approaches existing today are based on the structure-activity relationship premise, which states that biological activity of a molecule is strongly related to its structural or physicochemical properties. This work presents a novel approach to the automatic classification of chemical compounds by integrating semantic similarity with existing structural comparison methods. Our approach was assessed based on the Matthews Correlation Coefficient for the prediction, and achieved values of 0.810 when used as a prediction of blood-brain barrier permeability, 0.694 for P-glycoprotein substrate, and 0.673 for estrogen receptor binding activity. These results expose a significant improvement over the currently existing methods, whose best performances were 0.628, 0.591, and 0.647 respectively. It was demonstrated that the integration of semantic similarity is a feasible and effective way to improve existing chemical compound classification systems. Among other possible uses, this tool helps the study of the evolution of metabolic pathways, the study of the correlation of metabolic networks with properties of those networks, or the improvement of ontologies that represent chemical information.

## Author Summary

Among the existing systems capable of computationally comparing chemical compounds, the majority use only structural and physicochemical properties. However, with the emergence of ChEBI and other chemical compound databases, it has become feasible to create a system that can use the relevance of compounds in a biological context as well. This setting enables the distinction of molecules with different roles in nature but similar structures, or similar roles and different structures. ChEBI is organized as an ontology that classifies chemical compounds, which we use to derive a semantic similarity measure that reflects the biological relevance of molecules. In an effort to use as much information as possible, we introduce Chym, a system that integrates structural and semantic information in a single hybrid metric, and we show the accuracy of the system in three distinct classification problems, which consist in deciding whether a compound crosses the blood brain barrier, is a P-glycoprotein substrate or an estrogen receptor ligand. Chym outperforms the previous attempts to solve these three problems, with a maximum accuracy of 90.0%.

## Introduction

The recent publication of large-scale chemical information, made available by PubChem, ChEMBL and ChEBI, for instance, increased the focus of the scientific community on the problem of chemical comparison. With the amount of chemical data being published and produced today, it has become increasingly necessary to devise automatic systems capable of handling this information. The creation of an effective and accurate system that can compare and classify chemical compounds is useful in a number of different applications. For instance, it can help the understanding of the evolution of metabolic pathways, [1]; it can improve the information retrieval of disease, phenotype, and other models that contain references to chemical compounds; it enhances the study and development of pharmacophores [2], [3]; and it can also aid in toxicology, e.g. to estimate whether a given compound is or has the potential to be harmful to animals or humans without attempting a potentially harmful *in vivo* experiment [4].

**Figure 1:**
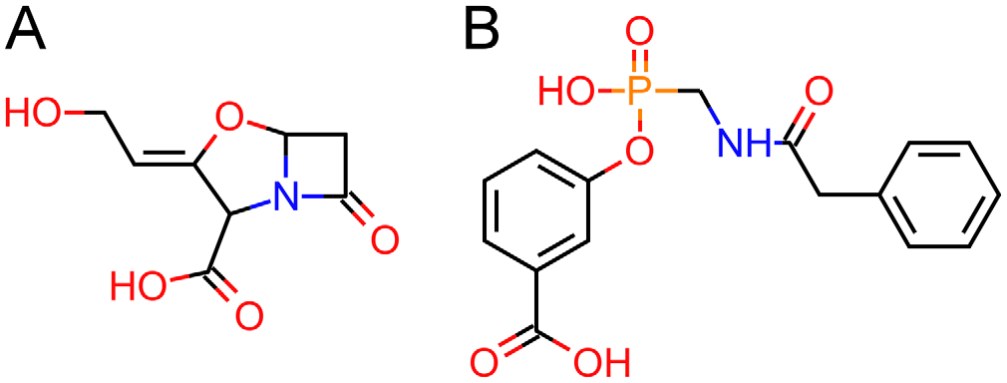
Chemical structure of two semantically related compounds. The two represented molecular structures, clavulanic acid (A) and 3-carboxyphenyl phenylacetamidomethylphosphonate (B), are different, and yet they both inhibit 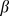-lactamase.

The best approaches existing today are based on the structure-activity relationship premise (SAR), which states that biological activity of a molecule is strongly related to its structural or physicochemical properties. While the existing methods prove that this assumption generally holds, it is not always true. For instance, while L-amino acids are used to synthesize proteins, their stereo-isomers, D-amino acids, are much less frequent in nature and their role is totally different [5]. From a biological point of view, they are distinct; however, to capture their structural differences, one needs to use three-dimensional methods (like optical methods [6]), and even with that consideration the structural similarity will be high, because both molecules have the same atoms and bonds. A possible solution involves simulating the docking between molecules and a protein pocket to determine whether they should interact *in vivo* [7], but this method needs the three-dimensional structure of the protein, and is only valid when the property of interest is caused by a protein binding mechanism (an example of a binary classification where no protein is involved is, although a simple one, the determination of liposolubility of chemical compounds). On the other hand, both clavulanic acid and 3-carboxyphenyl phenylacetamidomethylphosphonate are 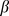-lactamase inhibitors, despite their different structures (see Figure 1). To address this problem, we propose the use of the *semantics* of a chemical compound in the context of biological relevance, which we used to improve the existing methods, through the development of a novel hybrid metric that takes into account both structural and semantic information. We dubbed the novel approach Chym, for Chemical Hybrid Metric. We extract semantic information from ChEBI, the Chemical Entities of Biological Interest ontology, an ontology containing more than 23,000 terms, which can be used at the base of semantic similarity [8]. Our proposal states that considering semantic similarity improves the performance of classification algorithms.

Most automatic classification methods implemented currently use either (i) the chemical structure as the foundation of the comparison [9], [10], or (ii) physicochemical properties like the molecular weight, the octanol-water partitioning coefficient etc. [11]–[14].

### Direct structure comparison methods

One of the main advantages of approach (i) is its ability to compare two or more molecules *on demand*, i.e., one can theoretically draw an arbitrary molecule and compare it to a whole database of structures without any prior knowledge about its function or properties.

There have been attempts to use graph comparison algorithms applied to the chemical structure of two molecules. One way of doing this is to restrict the similarity problem to the search for the maximum common sub-graph [15]. The general topology of the molecule can be used as the base of chemical similarity measures as well, where, for instance, a molecule can be represented as the matrix of the number of bonds between any two atoms and compared based on those matrices [16].

More often, though, structural similarity is calculated with the aid of fingerprints. A fingerprint, in this context, is a bitstring, a sequence of 0's and 1's, where each bit represents the presence or absence of a given feature or substructure. There are several ways to construct the fingerprint. For instance, for Daylight fingerprints, all the distinct linear fragments, up to a certain size, are identified from the graph and then converted into numbers 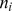 (usually, a hash function is applied to the fragment followed by a modulo function, effectively obtaining a number in the required range). The 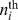 bits in the fingerprint are then set to 1 [17], [18]. Other methods assign a particular substructure to each one of the bits of the fingerprint. Two molecules can then be quickly compared based on the number of common bits in the fingerprints, for example, through the Jaccard-Tanimoto coefficient [19].

### Comparison from physicochemical properties

For approach (ii), one has to compute the describing properties (if possible), to gather them from literature or to conduct experiments to obtain them.

For example, in [11], the authors used Artificial Neural Network (ANN) and Support Vector Machine (SVM) to distinguish compounds capable of crossing the blood-brain barrier (**BBB**) from those that do not cross it. Each compound is described as a 9-dimensional vector, where each element is a physicochemical property of the molecule (molecular weight, volume, total surface area etc.). An ANN is composed of a number of artificial neurons (a conceptual object that receives several input values and combines them non-linearly to produce a single output) arranged in layers, where the first layer gets as input the descriptors of the molecule and the last layer outputs the classification; the SVM method consists of finding the hyper-surface that best separates the active compounds' vectors from the inactive compounds' [20].

In [2], the authors used a three-dimensional representation of molecules and applied an approach named “four-point pharmacophore”. This approach builds millions of descriptors, each being a different spatial arrangement of 4 features with the respective distances between them, and then determines whether the compound contains each of the descriptors, effectively constructing a bitstring which can be used like fingerprints, as previously described. In their work, the four-point pharmacophore model was used to predict whether compounds are substrates of the P-glycoprotein (**P-gp**). A SVM approach was also attempted on this set [21].

The work of [13] applies the concept of decision forests to predict whether a chemical compound binds to an **estrogen** receptor. A decision tree consists of several if-then statements, operating over the descriptors, which ultimately come together to create a tree with several branches. The last limbs of the tree classify the compound as active or inactive. A decision forest is then an ensemble of several decision trees, where each tree is constructed from the set of descriptors still not used in previous trees, so as to minimize the fraction of misclassifications, and the final output is a combination of the outputs of the trees [13].

Random forests also use decision trees as its basis, as shown by [12]. In their work, they used random forests to classify compounds as active or inactive in several sets, including the BBB, P-gp and estrogen sets above. Unlike the decision tree approach, however, the descriptors used in each tree are randomly drawn from the set of all descriptors, rather than drawn from the set of unused descriptors.

These previous works (as well as the present study) validate their approaches by using the comparison algorithms as classification systems and consistently report performance as the fraction of correctly classified compounds: 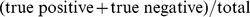. Table 1 presents the accuracy values obtained from those systems. To evaluate the effectiveness of our approach, we took the data of these previous studies and compared the outcome of our measure to those results.

**Table 1:** Performance of previous works.

Dataset	Classification system	Accuracy	Reference
BBB	Artificial Neural Networks	75.7%	[11]
	Random Forest	80.9%	[12]
	Support Vector Machines	81.5%	[11]
P-gp	Four-point Pharmacophore	62.7%	[2]
	Support Vector Machines	79.4%	[21]
	Random Forest	80.6%	[12]
estrogen	Decision Forest	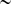 80%	[13]
	Random Forest	82.8%	[12]

This table summarizes the performance of several classification methods used on the BBB, P-gp and estrogen problems.

### Semantic properties

The semantic information of an object, i.e., its meaning in a predetermined context, is not easily handled by computers, mainly because meaning is mostly described in terms of natural language. For this reason, comparing the semantics of two objects (in this case, two chemical compounds), is not a straightforward task, and is only possible if the semantics of both objects are described under a common schema [22]. In this work, we used the ChEBI ontology (see below) to semantically describe chemical compounds, and under that common schema, we were able to derive a semantic similarity metric.

An ontology is a representation of terms and the relationship between them, and is usually visualized as a directed graph where nodes are the terms and the directed edges are the relationships [23]. A common type of relationship in ontologies is the “is a” relationship. It expresses the fact that one term's meaning subsumes the other's meaning, or, in other words, one term (the child) is a subclass of another term (the parent). Thus, some ontologies can be interpreted as directed acyclic graphs (DAG), where a term can have several parents and children; in such a graph, the deeper a term is, the more specific is its meaning. In the context of ontologies, a semantic measurement between two terms measures their proximity in the ontology. One of the simplest ways to compare two terms is to count the minimum number of relations that must be crossed to get from one compound to the other [24]. Another approach, used in DAGs, is to find the closest common ancestor of both terms; the distance between them is then the maximum number of relations from one of the two terms being compared to the common ancestor. It is worth noting that a measure can be a *distance* (as the terms get closer, the distance decreases) or a *similarity* (as the terms get closer, the similarity increases). Here we will consider only similarity measures.

In this work, we used both the ontology as a graph and a concept known as information content. The information content is an abstract concept that reflects the *specificity* of a particular object [25]. From information theory, the information content of an object can be evaluated as the negative logarithm of the probability of finding that object [24]. When calculating information content, it should be noted that a function is only meaningful if each term's occurrence contains all its children's occurrences too. In an ontology like ChEBI, this means that for more abstract terms the probability embraces many terms, decreasing its information content, which, in turn, reflects its low specificity. The probability function we will use is based on the number of pathways each compound participates in. The reason behind this choice is that counting the number of pathways gives a measure of specificity (compounds or chemical classes that are more specific will be found in less pathways), but it is not biased against the problems that Chym tries to solve.

## Results

To validate the effectiveness of Chym as a classification tool, we tested it on the sets presented in Table 1 and compared our results with the ones in that table. Since the results of chemical classification algorithms are usually reported in terms of accuracy (the fraction of correctly classified compounds), we report accuracy of Chym. However, for binary classification problems, Matthews Correlation Coefficient (MCC) is a better performance indicator [26]. Therefore, we use this coefficient as the main measure of Chym's performance.

### Sources of data sets

In the three sets retrieved from the previous works presented in the introduction, the compounds were listed by name only, with no information on structure. The first step in the assessment of Chym was, therefore, to translate that list of names into ChEBI identifiers. The task of getting the identifiers was accomplished by string matching techniques, since there was no structural information to make the search. We split the names into bags of words, where a word is a sequence consisting of only letters or only numbers, to determine whether two names refer to the same chemical entity. We used not only the preferred names of the compounds but also the synonyms stored in the ChEBI database. Only compounds present in the ontology and with a described molecular structure in the ChEBI database were considered. Because ChEBI is continually growing, we estimate that older compounds in the ontology are usually more correctly annotated and tend to have lower identifiers. So, in case of more than one possibility, we chose the lowest ChEBI id.

Since the ontology does not contain all the possible molecules, we were not able to get a full mapping between names and ChEBI compounds, which means that our sets were shorter versions of the original ones. We refer to our smaller sets as purged versions and denote them as BBB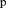, P-gp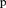 and estrogen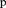. Table 2 shows the fraction of compounds in each of the three sets that are present in the ontology.

**Table 2:** Fraction of compounds in the ChEBI ontology.

Testing set	ChEBI coverage		
	active	inactive	overall
BBB	74/180	79/144	47.2%
P-gp	57/109	24/87	41.3%
estrogen	42/132	59/101	43.3%

Fraction of names found in the ChEBI ontology for each set of molecules. Coverage for active and inactive compounds is detailed.

The results of this table show a significant reduction in the size of all three sets after converting the names into ChEBI identifiers. Facing these values, we chose to directly compare our results only to the ones obtained with the blood-brain barrier, because (i) it is the set with higher percentage of ChEBI coverage, (ii) after purging, it remains the biggest set, and as such is more fit to be broken into testing and training sets without losing too much information, and (iii) it is the set with a more balanced distribution of active vs. inactive compounds. We will also apply Chym to the two other sets, but the analysis will not be as deep.

### Validation process

The BBB set is first described in [11], where the authors use an artificial neural network (ANN) and a support vector machine (SVM) to classify several chemical compounds as either able to cross the blood-brain barrier (active) or unable to do so (inactive). The paper showed that SVMs are more effective in this particular classification problem than ANNs (see Table 1). The set was further used by [12], where a random forest was grown to classify the compounds. The authors of this work showed the effectiveness of this system in several chemical compound sets, but the results obtained for the BBB set in particular were not better than the ones obtained with SVM. For this reason, we compared our results to the 81.5% accuracy reported by [11] (cf. Table 1). It is worth mentioning again that we report accuracy only for comparison purposes, but the real performance indicator should be MCC, which is also reported in the tables with the results.

In order to make an unbiased comparison between Chym and SVMs, we addressed the validation process in three steps, which were devised so that only one specification of the process changed in each step:

1. The SVM model described in [11] was used to replicate the results reported in that paper with the original set;
2. The same SVM model was used in our purged set, BBB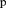;
3. Finally, we replaced the SVM model with our Chym approach.

It must be mentioned here that Chym is actually a collection of 24 metrics, each having a real parameter, 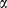, that balances the metric between structural and semantic information. We used 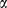 values from 0 to 1 in steps of 0.01, making a total of 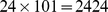 metrics (to understand the reason for these numbers, refer to the section Methods). The metric that yields the highest Matthews Correlation Coefficient is reported on the tables, alongside the MCC and accuracy values achieved with that same metric.

For the SVM approach, we retrieved the compounds' properties from the article as 9-dimensional vectors and used the SVMlight [27] software with a radial basis function kernel, as described in [11].

Moreover, to decrease the potential bias in our analysis, we implemented two different validation methods. The first one is a leave-multiple-out process, described in [11] and here dubbed “LMO25”. LMO25 follows this algorithm:

1. 25 active compounds and 25 inactive compounds are randomly removed from the set. They now form the testing set;
2. The remaining set is used to train the model;
3. The compounds in the testing set are classified according to the model learned in the previous step. Performance (as MCC and accuracy) is recorded;
4. Steps 1–3 are repeated 30 times, and an average of the performance indicators is recorded.

The second validation approach is 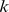-fold cross-validation, which is more widely used and well documented [28]. It starts by partitioning the compounds in the original set into 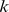 approximately equal-sized, stratified sets, meaning that the proportion of active inactive compounds is maintained in the partitions. Then each partition is used as testing group once, with the other 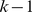 partitions being used to train the model. Accuracy and MCC values are recorded for each partition and averaged in the end of the 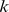 iterations. To remove the noise coming from the initial partition, we performed this method 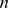 times and averaged the accuracy and MCC obtained. This validation approach was also applied to the P-gp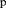 and estrogen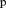 sets. We used 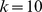 and 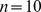 throughout the whole analysis [28].

The last step in the assessment of Chym was to predict some new active compounds in each of the three sets. We calculated an activity coefficient for all compounds in the ChEBI ontology annotated with a structure, based on the active compounds in the respective purged sets, and the best metric for each problem, and retrieved the ones whose coefficient was higher. For a discussion about the methods used to calculate this value, refer to section **Methods**.

### Performance

Table 3 shows the main results of the validation process, including the attempt to replicate the results of [11]. Given that we have 24 different metrics, each one tuned with a real parameter 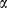, Chym had to select one of the possibilities. The best combination for this problem with the 10-fold cross-validation approach was FP3 fingerprint format with semantic similarity calculated for all the ontology with a simGIC method, with 29% of weight to structure and 71% to semantics (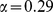). The same metric was pre-chosen for the LMO25 approach, even though Chym could have found that the best metric with this approach was not this one. The parameters of Chym's best metric (in this case FP3, simGIC, the whole ontology and the value of 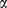) are explained in more detail in the Methodology section below. The results presented in the table show the superiority of Chym when compared with the SVM approach. Moreover, when we compare the two sections of the table with each other, it is possible to see that the validation method does not significantly affect the results. Since the 10-fold approach is more widely used, at least when compared to the LMO25, we performed the main analysis of our results with this method.

**Table 3:** Replication of the results of BBB.

Set	Approach	Validation method	Accuracy	MCC
BBB	SVM	LMO25	81.3%	0.630
BBB 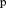	SVM	LMO25	73.8%	0.484
BBB 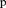	Chym	LMO25	89.6%	0.800
BBB	SVM	10-fold	81.2%	0.625
BBB 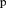	SVM	10-fold	74.1%	0.492
BBB 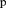	Chym	10-fold	90.0%	0.810

For the LMO25 method, the accuracy values are the mean of 30 experiments, as explained in the previous section. The Chym results were obtained for FP3 fingerprint format, simGIC semantic method using the entire ontology, and 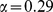.

In its second part, Table 3 shows the results of using 10-fold cross-validation instead of LMO25. Here we show that the accuracy of the SVM method used previously decreases significantly when some of the compounds in the set are removed. However, the same purged set can be used by Chym, and still achieve an accuracy 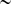
10% superior to the one originally reported, with an associated Matthews Correlation Coefficient increase of almost 0.2 units. One possible explanation for this is the effect of the retention of chemotypes. Information on the chemotypes is implicitly contained in the ontology, which may buffer the effect of removal of individual molecules. It would be interesting to make an analysis of chemotypes, for example through Murcko scaffolds [29], [30]. However, the fact that the data sets were retrieved by name and not by structure invalidates this approach. But the SVM approach's accuracy decreases 6–8% when used on the purged set, which seems to suggest that the chemotype retention is not prominent.

Table 4 shows the performance of Chym when applied to the data sets used. The “Chym parameters” column specifies the parameters of the best metrics in terms of which fingerprint format, semantic method, ontology and 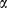 value are best suited for that set (cf. Table S1, Table S2, Table S3 in the Supporting Material, available online for the results obtained for all metrics). This table reinforces the prediction power of Chym, since its performance with the P-gp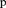 and estrogen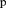 sets, which are also about 60% smaller than the original ones, is still higher than (for the P-gp set) or comparable to (for the estrogen set) the value obtained with the random forest approach, the best method applied so far to those sets (cf. Table 1). Although there does not seem to be any improvement in the estrogen problem, we must underline that we have used a smaller set, and we believe the performance would increase with a more complete ChEBI ontology. On the other hand, using the values reported in the work about random forests [12], it is possible to recalculate the MCC value obtained with that method, 0.647. The value of 0.673 achieved with Chym represents a slight increase.

**Table 4:** Results of the classification system derived from the Chym comparison method.

Set	Chym			Best previous attempt		
	Parameters	MCC	Accuracy	Approach	MCC	Accuracy
BBB 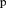	FP3, simGIC, all, 0.29	0.810	90.0%	SVM	0.628	81.5%
P-gp 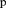	FP4, simUI, role, 0.72	0.694	87.3%	Random Forests	0.591	80.6%
estrogen 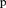	FP4, simGIC, role, 0.45	0.673	82.6%	Random Forests	0.647	82.8%

Chym parameters are “fingerprint format, semantic method, branch of the ontology used, 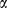
”. The validation process used was 10-fold. Matthews Correlation Coefficient values reported here was not directly retrieved from the papers where the attempts are described, but were estimated based on the values of true positives, false positives, true negatives and false negatives given in those papers.

**Figure 2:**
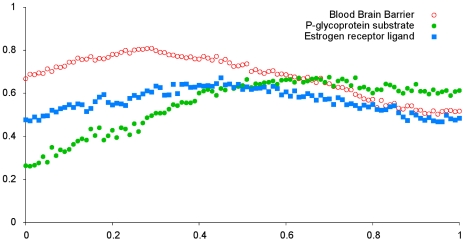
The effect of the parameter 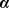 in the performance of Chym. For each dataset, the best metric was stripped of the 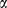 parameter. This incomplete metric was completed with all values of 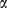 and then each one was used to determine performance. The figure shows the variation of performance (as measured by the Matthews Correlation Coefficient) against the value of 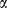. There is a maximum in the plot for every dataset, consisting of the best metric: 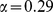, 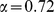, 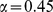 for the BBB (red open circles), P-gp (green closed circles) and estrogen (blue closed squares) datasets respectively).

Table 5 and Figure 2 show the MCC of three Chym systems against 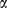 values. For each set, the parameters used with the Chym system are the ones which reached maximum accuracy for some value of 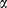. It is visible that, in the three Chym systems, the accuracy starts by increasing at first, reaching a maximum, and decreasing again. This shows that using the hybrid measure is better than using only purely structural or semantic metric. When this same analysis is applied to other Chym parameters, we can observe the same behavior, which confirms the idea that, even if one system is not very accurate, the crossing of structural and semantic information increases the prediction power of Chym.

**Table 5:** The effect of the alpha parameter in Chym's performance as measured by the Matthews Correlation Coefficient.

Alpha	BBB 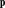	P-gp 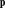	estrogen 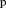
0.0	0.66837	0.47723	0.26418
0.1	0.74508	0.54799	0.33957
0.2	0.78206	0.54634	0.42900
0.3	**0.79941**	0.63492	0.50817
0.4	0.75904	**0.63774**	0.60167
0.5	0.73267	0.61939	0.63670
0.6	0.68652	0.60764	0.66318
0.7	0.64528	0.57530	**0.67470**
0.8	0.57281	0.54896	0.60161
0.9	0.52186	0.49979	0.64252
1.0	0.51764	0.48429	0.61333

The Chym parameters used are the ones in Table 4, except that instead of a single 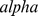 value, we present results for several values. Validation was performed with a 10-fold approach. Bold values are the maximum for each column.

Finally, Table 6 shows the most active ChEBI compounds, as defined by the activity coefficient, retrieved for each problem. In each problem, we retrieved all the ChEBI compounds in the ontology and with a molecular structure (more than 15000) and ranked them by activity coefficient. The table reports the first three whose classification has been previously determined in a publication and shows that those compounds are, in fact, active compounds (they cross the blood-brain barrier, are substrates to P-glycoprotein or ligands to the estrogen receptor), which also contributes to the idea that the Chym method is effective. The entire ranked lists of compounds are available as Table S4, Table S5, Table S6.

**Table 6:** The activity coefficients of the most active compounds in ChEBI when compared to the active compounds in each set.

Set	Rank	Compound		Coefficient	Ref.
		ID	Name		
BBB 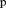	1	50931	(Z)-chlorprothixene	0.289	[44]
BBB 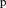	2	51137	mianserin	0.280	[45]
BBB 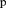	3	251412	adinazolam	0.279	[46]
P-gp 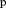	7	53290	(S)-donepezil	0.373	[47]
P-gp 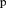	15	31181	aklavinone	0.368	[48]
P-gp 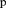	16	48723	(-)-lobeline	0.366	[49]
estrogen 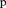	2	27917	luteone	0.277	[50]
estrogen 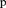	4	5262	galangin	0.274	[51]
estrogen 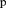	5	50399	3′,4′,7-trihydroxyisoflavone	0.274	[52]

For each compound, a reference showing that the compound is indeed active is given. The thresholds for each problem, as determined by the algorithm detailed in the Methodology section, are 0.243 (BBB), 0.272 (P-gp) and 0.231 (estrogen).

## Discussion

The work presented in this paper shows compelling evidence that using semantic information in chemical classification algorithms improves their performance. To show that, we used three sets of compounds previously described and used as input in other classification methods. On those sets, Chym achieves higher performance for class prediction when compared to previously existing methods, with Matthews Correlation Coefficient as high as 0.810, corresponding to an accuracy of 90.0%. Parallel to this result, we also showed that the use of a hybrid metric that uses both structural and semantic information is better suited for this kind of problems than a system which uses only one of these types of information. Some issues should, however, be discussed in order to complete the analysis of this tool.

The properties that are relevant to decide whether a molecule should be classified as active or inactive depend obviously on the problem being solved. As such, the best metric for a problem is not necessarily the same for other problems. Thus, selecting the best metric is not much different than selecting the appropriate descriptors for SVM, random forest or the other approaches presented before. While it may be argued that the value of 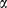 is inherent to Chym's method, it does reflect the relative amount of structural and semantic information that must be used to correctly classify compounds. Choosing the appropriate value for this and the other parameters can be seen, from the point of view of usage, as a task similar to choosing the appropriate descriptors that better reflect the important characteristics of the molecules (those that yield the best results). For instance, as specified in Table 4, while the FP3 fingerprint format is good at detecting some substructures that are important in the BBB problem, it misses the relevant structures in the other problems. Furthermore, the BBB problem is better solved with a stronger focus on the semantic information, and the 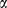 value of the best metric reflects this, as evident in Figure 2.

On another note, our high performance could be due to a possible term in the ontology that classified compounds as able to cross the blood-brain barrier, as substrates to the P-glycoprotein or as estrogen receptor ligands. Admittedly, if there were such terms in the ontology, Chym would be biased and would report high accuracy values because it would be using the information it was trying to validate as a means to prove its effectiveness. As it turns out, no term in the ontology refers to the words “brain”, “barrier”, “P-glycoprotein” or “permeability” (the meaning of the P in P-glycoprotein). “Estrogen receptor” appears twice, in “estrogen receptor modulator” and “estrogen receptor antagonist”, but these two terms have only a total of 5 descendants in the ontology, and none of them is present in the set estrogen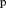. This fact suggests that Chym can be used in many classification and similarity problems, even if they are not well represented in the ontology.

The reason for this fact is that, although the information to solve the classification problem is not explicitly stated in the ontology, the proximity of terms in the ontology (their semantic similarity) is a good indicator that they should behave similarly. For instance, both the compounds ChEBI:8069, phenobarbital, and ChEBI:49575, diazepam, cross the blood brain barrier. Moreover, they share many of their ancestors. Their semantic similarity, as measured with a simGIC method in the whole ontology, is 0.324, and their structural similarity, as measured with the FP3 format, is 0.667. With an 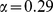 (these are the parameters chosen for the BBB problem, cf. Table 4), this results in a similarity of 0.423, well above the mean similarity between the active compounds in the BBB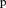 set, 0.238. The compounds are both annotated as sedative drugs, and Chym was then able to determine that ChEBI:51137, mianserin, another sedative drug, also crosses the BBB (see Table 6).

Still in respect to the results presented in Table 6, a further analysis showed that ChEBI:5078, flavonol, was ranked 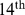 in the list of estrogen receptor ligands (activity coefficient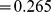, higher than the threshold calculated for that problem), but [31] showed that this compound is not an estrogen receptor ligand. However, the class of compounds named flavonoids, into which flavonol is classified, is known to contain several compounds that bind to the estrogen receptor [32], [33]. Moreover, this compound shares most of galangin's ancestry, 58 common ancestors out of 61 total ancestors (galangin is also on that table). This means that the ChEBI ontology is not yet able to differentiate between these two compounds, and so it produces a false positive. As a matter of fact, the similarity between these two compounds in the metric chosen for the estrogen problem is 0.716, while the mean similarity between all the active compounds is 0.216, demonstrating that ChEBI assigns high similarity to these molecules.

As discussed in the Methodology, the ChEBI ontology contains three partially overlapping branches. One concern raised by this fact is that the molecular structure more or less reproduces the structural information used in the first part of the metric. Although the information being used is indeed the same, the ontology explores the structural properties from a totally new perspective (namely, a semantic perspective), that would be otherwise unusable in a similarity measure: purely structural comparison methods are probably unable to use the fact that both glucose and fructose are monosaccharides to compare them. So, even if there seems to be a duplication of information, the different approaches used yield similarity values that can be combined to produce a more robust score (as Chym does).

Another concern raised about the use of ChEBI ontology is the subatomic branch. This branch was never chosen by itself as the best branch of the ontology, which is not surprising, for two reasons. First, it is not much richer than the molecular structure or role branches, since only 35 ChEBI terms are unique to this branch of the ontology. Secondly, each of these 35 terms is either an ancestor to all chemical compounds used in the input set (as happens with electron, for instance, which is part of the atom, which is part of every molecular structure) or ancestor to none of the chemical compounds (photon, for instance). This means that this branch does not offer any kind of resolution.

However, like any other classification algorithm, Chym has its limitations. The most important drawback of this method is that it can only compare structures that are annotated in the ChEBI ontology. Of course that any chemist or other scientist wishing to use Chym may annotate the compound they are trying to study in ChEBI by creating a “non-official” node. There is, however, a large number of classes, which could potentially introduce a difficulty in selecting the most appropriate position for the compound; this annotation is also unfeasible for a large number of compounds. This severely impairs applications like drug discovery, or toxicology analysis.

In spite of this limitation, Chym introduces the comparison of chemical compounds through their semantics, which is an important technique that can be used in projects where comparison and or classification of known chemical compounds is needed. One instance of such project is the search for a possible correlation between strains of bacteria and their virulence. One could be interested in determining differences in metabolic networks of said strains and compare the differences with the different amount of virulence of those strains; the comparison of metabolic networks would benefit from the metrics explored here. Other applications include the comparison of models, for instance models of diseases containing references to molecules responsible for the disease or to drugs known to improve the condition of patients. On the other hand, the semantic similarity applied to ChEBI (developed and explored in this work) can also be useful in ontology managing, as happens in GO [34], where semantic similarity is used to automatically annotate other molecules in the ontology and automatically improve the ontology. This would in turn be useful in information retrieval and automatic reasoning methodologies.

In the future, it would be interesting to try other hybrid metrics, especially other structural comparison algorithms. For instance, since SVM and random forests seem to perform well, perhaps a system where the structural part of the comparison is done through one of these methods would outperform the actual version of Chym.

## Methods

In order to develop and validate our hybrid similarity for chemical compounds, the **C**hemical **hy**brid **m**etric (Chym), we built a model based both on fingerprints and on the semantic similarity measures developed for the Gene Ontology (GO) [35].

### Structural similarity

To calculate the structural similarity between two molecules, we need a representation of their structures. Because ChEBI contains a list of structures in SMILES, MDL and InChI chemical file formats, these are the formats used. For each distinct molecule, we prefer a SMILES representation of the structure. If one does not exist, we use MDL. The rationale for this choice is the wide use of SMILES over MDL. InChI was not used since every molecule with a structure in this format had at least one of the other formats as well.

For each structure, three fingerprints were calculated. These formats were computed with the OpenBabel software [36], [37], and as such we used the names and files provided by it:

**FP2** All non-branched (linear or possibly circular) fragments of up to 7 atoms are calculated from the initial structure. Each fragment is assigned a number from 0 to 1020 by means of a hash function and the corresponding bit in the fingerprint is set to 1.
**FP3** The molecule is analyzed and, if a specific pattern is identified, its corresponding bit in the fingerprint is set. The patterns are defined in a file that is part of the OpenBabel software.
**FP4** This format is conceptually the same as the FP3 format but with different patterns, which are defined in a different file.

Given two molecules and the corresponding fingerprints 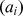 and 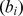, the similarity score between them is calculated according to the Jaccard-Tanimoto coefficient [17], [38], [39]:
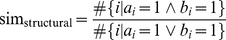
(1)where 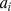 and 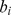 are the 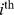 bit in each of the fingerprints. Obviously, comparison of fingerprints is only valid if the fingerprints were obtained by the same method. This equation is valid only if the denominator is different from 0. It was verified that all fingerprints calculated had at least one bit set to 1, thus making the denominator always positive.

From equation 1, it can be seen that the structural similarity will run from 0, when no bit is 1 for both molecules (total disparity), to 1, when the 1-bits in the two molecules are the same (equal fingerprints).

### Semantic similarity

Following the application of semantic measures for the GO [35], we developed a similar approach but instead of proteins, we work with chemical compounds. As has been stated above, there are a number of ways to measure semantic similarity based on an ontology. We chose to use the same ones as [35]. In the next paragraphs, consider 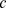, 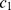 and 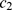 as chemical compounds and 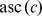 as the set of ancestors of the chemical compound 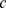, including 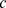 itself.

**simUI** is a graph-based measure, which means that it considers the compounds and all their ancestors in the graph of the ontology. It is defined as follows [40]:
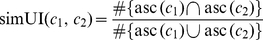
(2)


It is known, however, that for ontologies where term specificity is not well correlated with term depth, methods based on information content (IC) are preferable [35]. Let 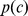 be the frequency of usage of the term 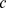 in some corpus. The information content of a term can be given by [41]:
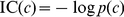
(3)Intuitively, equation 3 means that a very frequent term conveys less information and vice-versa. Notice that the frequency of a term 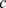 subsumes the frequency of the terms that derive from 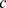. This means that the frequency of the term amino acid includes the frequency of terms L-serine or carnitine, a 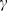-amino acid. Therefore, less specific terms are less informative. Chym makes use of this equation, where the terms are the nodes of the ChEBI ontology, *viz* chemical compounds or chemical classes.

**simGIC** is a combination of the graph-based simUI metric with the information content properties of compounds. The concept behind the equation is the same as the one behind simUI, but now each ancestor is weighted according to its information content, which reflects its specificity. simGIC is calculated through equation 4 [35].
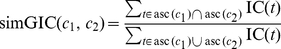
(4)


It is worth underlining here that the concept of information content is just a method to give weight to the compounds in the ontology. If two compounds share many ancestors, simUI will attribute a high similarity between them, but, for example, if most of those ancestors are unspecific, the similarity should be lowered accordingly; by weighting the ancestors, simGIC achieves this effect. For example, compounds ChEBI:17802, pseudouridine, and ChEBI:31747, kanosamine, share 30 or their 37 ancestors, but the most specific of those is ChEBI:23008, carbohydrate, already a very abstract term in the ontology. simGIC takes into account this fact. Considering the similarity values between all pairs of compounds that appear in the corpus at least once, the mean similarity measured with simUI is 0.431 and the mean similarity with simGIC is 0.048. Those two compounds share a simUI similarity value of 0.811, about twice the mean value, but by weighting the ancestors, simGIC assigns a similarity of 0.023, about half of the mean value.

For both metrics, the similarity value is between 0 and 1 because an intersection of two sets is always a subset of their union.

### Hybrid metric

Until this point, we presented two orthogonal metrics to measure the similarity between two chemical compounds. Our intent, however, is to join them together to produce a hybrid metric that takes into account both structural and semantic information.

Since both measures explained above always fall in the closed interval 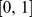, we propose the following definition for our hybrid similarity:
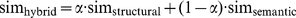
(5)where 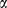 is a real number from 0 to 1. When 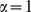, the identity degenerates into pure structural similarity and with 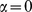, into pure semantic similarity.

### Chym approach to classification

One of the possible uses of Chym is the application of this similarity metric to classify compounds. Ideally, we want to be able to get a set of chemical compounds that possess a common property as input, and then determine whether other chemical compounds also possess that property. This is also the approach used in SVM and random forests, for example, where the input serves as a training set that is used to create a classification model. In Chym, the model consists of a threshold that is used to decide whether a compound is active or inactive.

Given a training set of compounds, some sharing a common property (which we call *active* compounds), and some lacking that property (*inactive* compounds), the following algorithm is used to predict whether a compound in the validation set is active or inactive:

1. Within the training set, compare each compound with all active compounds. The comparison of an active compound with itself is excluded, since this value (which is always 1) could introduce a bias into the rest of the algorithm.
2. For each of the compounds in the training set, determine its *activity coefficient*, which is the unweighted average of the results in step 1. A compound will be classified as active if its activity coefficient is above a threshold, which still needs to be calculated.
3. Determine the *threshold of activity*, 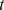. To do this, Chym uses all the coefficients calculated in step 2 as potential thresholds, and classifies the compounds in the training set as active or inactive accordingly. The coefficient that minimizes the number of misclassifications in the test set is chosen. This ends the training step.
4. For all compounds in the validation set, Chym calculates their activity coefficient as the average of similarities between the compound and all active compounds in the training set, and classifies it as active if the activity coefficient is greater than or equal to the threshold of activity 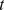, and as inactive otherwise.

From the algorithm above, it can be seen that the inactive compounds are only used to adjust the value of the threshold, while the active compounds are used both in the adjustment of that value and in the determination of the activity coefficient of the validation compounds.

### Data sources

**Chemical Entities of Biological Interest** (ChEBI) is a freely available database of small molecular entities (distinct isotopes, atoms, ions, molecules etc.). These entities may be products of nature or synthetic products used to intervene in biological processes [8].

The ontology also includes classes of molecular entities and partial molecular entities, enabling ChEBI to be organized as an ontology, structuring molecular entities into classes and defining the relations between them. Several relationship types exist in ChEBI, with a number of them reciprocal in nature. The ontology is subdivided into three separate sub-ontologies:

- **Molecular structure**, in which the entities are classified according to composition and structure.
- **Role**, in which entities are classified according to their role within a biological context.
- **Subatomic particle**, which classifies particles smaller than atoms.

As of the time of the computations (January 2010, release 64), the graph of this ontology contained 23,545 nodes representing chemical compounds, which represents approximately 4% of the whole ChEBI database. As stated above, some terms are not chemical compounds but parts of compounds, such as functional groups, that make the ontology structure possible. Also, for each individual chemical compound, there may be several identifiers, which come from different annotations that were later identified as the same compound.

Chym's branches are partially overlapping. For instance, the term glucose is classified as a molecular structure, as having the role of macronutrient and as having part electron, which means that it is present in three branches. Including glucose, 21676 nodes (92%) are part of the three branches.

Besides the ontology, the ChEBI database is enriched with an extensive list of synonyms and manually curated cross-references to other non-proprietary databases, as well as a list of chemical structures.

**Kyoto Encyclopedia of Genes and Genomes** (kegg) is a collection of databases categorized into systems information, genomic and chemical information. The different kegg databases are highly integrated in an effort to constitute a computer representation of the biological system [42].

One of the main components of kegg is the kegg pathway database, which contains a collection of graphical representations of known pathways. Each metabolic entry integrates information from other databases in kegg, such as the intervening enzymes (kegg enzyme), chemical reactions (kegg reaction) and chemical compounds present in the pathway (kegg compound).

kegg compound is a chemical structure database for metabolic compounds and other chemical substances that are relevant to biological systems. We use entries in the kegg compound database as annotations of the compounds present in the metabolic pathways (kegg pathway entries). The existence of a mapping between ChEBI and kegg compound allows us to integrate information from both databases.

### Implementation

The methods used to structurally compare compounds are implemented by the software, OpenBabel [36], [37]. We used version 2.2.3, which was downloaded and installed on December 2009.

The semantic similarity was not as straightforward. As in [43], we had to reorganize the ChEBI ontology so that it could fulfill our purposes. All cyclic relationships (“*is tautomer of*” etc.) were removed, and the other relationships were merged into a single “*is a*”-like relationship. Also, ChEBI identifiers pointing to the same chemical compounds were merged into a single node. Thus, we produced three independent DAGs, one for each branch of the main ontology, and a forth DAG resulting from merging the other three. With this modification, we can directly calculate simUI similarities with equation 2.

To calculate the IC-based metric (simGIC), we had to find a corpus where the compounds are referenced. We chose kegg pathway because it is not connected to any of the problems solved by Chym. This has the advantage of avoiding a potential bias that could boost Chym's results. To map a ChEBI identifier into a kegg identifier, we used the ChEBI cross-references. Sometimes, however, these references were ambiguous (one ChEBI id pointing to two or more kegg compound ids). For this reason, whenever a ChEBI id 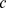 had more than one kegg compound reference, we used them all to determine the number of pathways in which 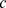 participates. With this corpus, the value of 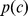 from equation 3 is the fraction of pathways where the compound 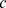 or any of its descendants appear.

Since there are 3 fingerprint formats, and semantic similarity can be calculated based on 4 different DAGs and with 2 different methods, the approach we are presenting here is able to use 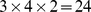 different similarity metrics, each with a real parameter 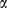.

## Supporting Information

Table S1 Performance indicators for every metric used by Chym, when solving the BBB problem. The table is sorted so that the metric with higher Matthews Correlation Coefficient appears first in the list.
(0.19 MB TXT)

Table S2 Performance indicators for every metric used by Chym, when solving the Pgp problem. The table is sorted so that the metric with higher Matthews Correlation Coefficient appears first in the list.
(0.19 MB TXT)

Table S3 Performance indicators for every metric used by Chym, when solving the estrogen problem. The table is sorted so that the metric with higher Matthews Correlation Coefficient appears first in the list.
(0.19 MB TXT)

Table S4 Activity coefficient of every compound in the ChEBI ontology, when the dataset from the BBB problem is used to train Chym. Only compounds with a structure were considered.
(0.66 MB TXT)

Table S5 Activity coefficient of every compound in the ChEBI ontology, when the dataset from the Pgp problem is used to train Chym. Only compounds with a structure were considered.
(0.66 MB TXT)

Table S6 Activity coefficient of every compound in the ChEBI ontology, when the dataset from the estrogen problem is used to train Chym. Only compounds with a structure were considered.
(0.66 MB TXT)
